# Ethyl 2-[(*Z*)-2-cyano­imino-1,3-thiazolidin-3-yl]acetate

**DOI:** 10.1107/S1600536808015663

**Published:** 2008-06-07

**Authors:** Bing Xie

**Affiliations:** a Henan Provincial Key Laboratory of Surface and Interface Science, Zhengzhou University of Light Industry, Zhengzhou 450002, People’s Republic of China

## Abstract

In the title mol­ecule, C_8_H_11_N_3_O_2_S, the puckering amplitude of the thia­zolidine ring is *q*_2_ = 0.3011 (5) Å and the conformation is an envelope. There are weak inter­molecular C—H⋯O inter­actions which stabilize the crystal structure.

## Related literature

For the crystal structures of related compounds, see: Dai *et al.* (2007 ▶). For details of the biological activities of thia­zolidine-containing compounds, see: Iwata *et al.* (1988 ▶). For bond-length data, see: Allen *et al.* (1987 ▶). For puckering amplitude definitions, see: Cremer & Pople (1975 ▶). For conformation definitions, see: Duax *et al.* (1976 ▶).
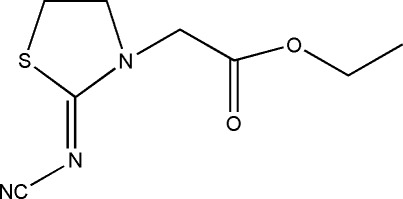


## Experimental

### 

#### Crystal data

C_8_H_11_N_3_O_2_S
*M* *_r_* = 213.26
Monoclinic, 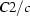

*a* = 30.862 (6) Å
*b* = 4.9376 (10) Å
*c* = 14.067 (3) Å
β = 105.09 (3)°
*V* = 2069.7 (7) Å^3^
*Z* = 8
Mo *K*α radiation
μ = 0.29 mm^−1^
*T* = 293 (2) K
0.34 × 0.21 × 0.15 mm

#### Data collection

Rigaku R-AXIS RAPID IP area-detector diffractometer
Absorption correction: multi-scan (*ABSCOR*; Higashi, 1995 ▶) *T*_min_ = 0.907, *T*_max_ = 0.958
7488 measured reflections
1826 independent reflections
1491 reflections with *I* > 2σ(*I*)
*R*_int_ = 0.045

#### Refinement

*R*[*F* ^2^ > 2σ(*F* ^2^)] = 0.039
*wR*(*F* ^2^) = 0.117
*S* = 1.10
1826 reflections
128 parameters
H-atom parameters constrained
Δρ_max_ = 0.25 e Å^−3^
Δρ_min_ = −0.31 e Å^−3^

### 

Data collection: *RAPID-AUTO* (Rigaku, 2004 ▶); cell refinement: *RAPID-AUTO*; data reduction: *RAPID-AUTO*; program(s) used to solve structure: *SHELXTL* (Sheldrick, 2008 ▶); program(s) used to refine structure: *SHELXTL*; molecular graphics: *SHELXTL*; software used to prepare material for publication: *SHELXTL*.

## Supplementary Material

Crystal structure: contains datablocks I, global. DOI: 10.1107/S1600536808015663/hg2405sup1.cif

Structure factors: contains datablocks I. DOI: 10.1107/S1600536808015663/hg2405Isup2.hkl

Additional supplementary materials:  crystallographic information; 3D view; checkCIF report

## Figures and Tables

**Table 1:** Hydrogen-bond geometry (Å, °)

*D*—H⋯*A*	*D*—H	H⋯*A*	*D*⋯*A*	*D*—H⋯*A*
C2—H2*C*⋯O2^i^	0.97	2.56	3.284 (3)	132
C4—H4*B*⋯O2^ii^	0.97	2.50	3.431 (3)	162

Symmetry codes: (i) 
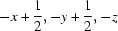
; (ii) 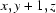.

**Fig. 1.**
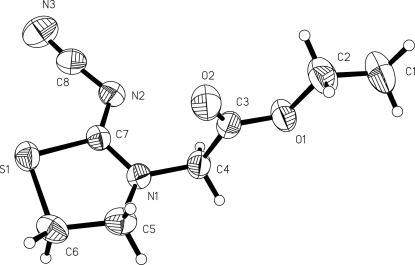
The molecular structure of (I), with atom labels and 40% probability displacement ellipsoids for non-H atoms.

## supplementary crystallographic information

### Comment

Thiazolidine is an important kind of group in organic chemistry. Many compounds containing Thiazolidine groups possess a broad spectrum of biological activities (Iwata *et al.*, 1988). Here, we report the crystal structure of (I).

In (I) (Fig. 1), all bond lengths are normal (Allen *et al.*, 1987) and in a good agreement with those reported previously (Dai *et al.*, 2007). The plane I (C7/C8/N1–N3/S1) makes the dihedral angles of 86.11 (3)° with ethyl acetate group (C1–C4/O1/O2). The Cremer & Pople (1975) puckering amplitude of the thiazolidine ring is q2 = 0.3011 (5) Å. According to Duax *et al.* (1976), the conformation is an envelope with a local pseudo-mirror passing through C6 and the mid-point of the N1—C7 bond. There are some weak C—H···O intermolecular interactions (see Table 1) which stabilize the title structure.

### Experimental

A solution of (*Z*)-(thiazolidin-2-ylideneamino)formonitrile 1.27 g (10 mmol) and sodium hydride 0.3 g dissolved in anhydrous acetonitrile (20 ml), and dropwise added over a period of 10 min to a solution of ethyl 2-chloroacetate 1.23 (10 mmol) in acetonitrile (10 ml) at 273 K. The mixture was stirred at 353 K for 3 h. The solvent was removed and the residue was purified by recrystall from ethanol to give I as a white solid (1.92 g, 90%). Single crystals suitable for X-ray measurements were obtained by recrystallization from ethanol at room temperature.

### Refinement

H atoms were positioned geometrically and refined using a riding model, with C—H = 0.96 or 0.97 Å, with *U*_iso_(H) = 1.2 times *U*_eq_(C) and 1.5 times *U*_eq_(C) for the methyl H atoms.

### Crystal data

**Table:**

C_8_H_11_N_3_O_2_S	*F*_000_ = 896
*M**_r_* = 213.26	*D*_x_ = 1.369 Mg m^−^^3^
Monoclinic, *C*2/*c*	Mo *K*α radiation λ = 0.71073 Å
Hall symbol: -C 2yc	Cell parameters from 1021 reflections
*a* = 30.862 (6) Å	θ = 2.9–26.4º
*b* = 4.9376 (10) Å	µ = 0.29 mm^−^^1^
*c* = 14.067 (3) Å	*T* = 293 (2) K
β = 105.09 (3)º	Block, colourless
*V* = 2069.7 (7) Å^3^	0.34 × 0.21 × 0.15 mm
*Z* = 8	

### Data collection

**Table:**

Rigaku R-AXIS RAPID IP area-detector diffractometer	1826 independent reflections
Radiation source: rotating anode	1491 reflections with *I* > 2σ(*I*)
Monochromator: graphite	*R*_int_ = 0.045
*T* = 293(2) K	θ_max_ = 25.0º
ω oscillation scans	θ_min_ = 3.0º
Absorption correction: multi-scan(ABSCOR; Higashi, 1995)	*h* = −36→36
*T*_min_ = 0.907, *T*_max_ = 0.958	*k* = −5→5
7488 measured reflections	*l* = −16→15

### Refinement

**Table:**

Refinement on *F*^2^	Hydrogen site location: inferred from neighbouring sites
Least-squares matrix: full	H-atom parameters constrained
*R*[*F*^2^ > 2σ(*F*^2^)] = 0.039	*w* = 1/[σ^2^(*F*_o_^2^) + (0.0531*P*)^2^ + 1.3476*P*] where *P* = (*F*_o_^2^ + 2*F*_c_^2^)/3
*wR*(*F*^2^) = 0.117	(Δ/σ)_max_ < 0.001
*S* = 1.10	Δρ_max_ = 0.25 e Å^−^^3^
1826 reflections	Δρ_min_ = −0.31 e Å^−^^3^
128 parameters	Extinction correction: SHELXTL (Sheldrick, 20018), Fc^*^=kFc[1+0.001xFc^2^λ^3^/sin(2θ)]^-1/4^
Primary atom site location: structure-invariant direct methods	Extinction coefficient: 0.0039 (9)
Secondary atom site location: difference Fourier map	

### Special details

**Table:**

Geometry. All e.s.d.'s (except the e.s.d. in the dihedral angle between two l.s. planes) are estimated using the full covariance matrix. The cell e.s.d.'s are taken into account individually in the estimation of e.s.d.'s in distances, angles and torsion angles; correlations between e.s.d.'s in cell parameters are only used when they are defined by crystal symmetry. An approximate (isotropic) treatment of cell e.s.d.'s is used for estimating e.s.d.'s involving l.s. planes.
Refinement. Refinement of *F*^2^ against ALL reflections. The weighted *R*-factor *wR* and goodness of fit *S* are based on *F*^2^, conventional *R*-factors *R* are based on *F*, with *F* set to zero for negative *F*^2^. The threshold expression of *F*^2^ > σ(*F*^2^) is used only for calculating *R*-factors(gt) *etc*. and is not relevant to the choice of reflections for refinement. *R*-factors based on *F*^2^ are statistically about twice as large as those based on *F*, and *R*- factors based on ALL data will be even larger.

### Fractional atomic coordinates and isotropic or equivalent isotropic displacement parameters (Å^2^)

**Table:**

	*x*	*y*	*z*	*U*_iso_*/*U*_eq_	
S1	0.462350 (18)	0.24900 (11)	0.03972 (4)	0.0520 (2)	
O1	0.27777 (5)	0.6328 (3)	−0.12161 (13)	0.0627 (5)	
O2	0.31720 (5)	0.3188 (3)	−0.02097 (14)	0.0670 (5)	
N1	0.39666 (6)	0.5821 (3)	−0.01316 (12)	0.0453 (4)	
N2	0.40242 (6)	0.2991 (4)	−0.13944 (13)	0.0514 (5)	
N3	0.44256 (9)	−0.0586 (5)	−0.20421 (18)	0.0847 (7)	
C1	0.19858 (10)	0.6384 (8)	−0.1823 (3)	0.0959 (10)	
H1A	0.1712	0.5457	−0.1828	0.144*	
H1B	0.2017	0.6507	−0.2483	0.144*	
H1C	0.1979	0.8173	−0.1559	0.144*	
C2	0.23648 (8)	0.4890 (6)	−0.1212 (2)	0.0781 (8)	
H2B	0.2373	0.3074	−0.1471	0.094*	
H2C	0.2335	0.4751	−0.0545	0.094*	
C3	0.31533 (7)	0.5240 (4)	−0.06723 (16)	0.0498 (5)	
C4	0.35509 (7)	0.6959 (4)	−0.07168 (18)	0.0525 (6)	
H4A	0.3565	0.7112	−0.1396	0.063*	
H4B	0.3513	0.8765	−0.0479	0.063*	
C5	0.40994 (8)	0.6115 (5)	0.09383 (16)	0.0553 (6)	
H5A	0.3904	0.5060	0.1235	0.066*	
H5B	0.4084	0.7998	0.1122	0.066*	
C6	0.45751 (8)	0.5090 (5)	0.12722 (15)	0.0557 (6)	
H6A	0.4634	0.4343	0.1931	0.067*	
H6B	0.4786	0.6545	0.1273	0.067*	
C7	0.41698 (6)	0.3814 (4)	−0.04819 (14)	0.0413 (5)	
C8	0.42527 (8)	0.1075 (5)	−0.17063 (16)	0.0572 (6)	

### Atomic displacement parameters (Å^2^)

**Table:**

	*U*^11^	*U*^22^	*U*^33^	*U*^12^	*U*^13^	*U*^23^
S1	0.0464 (4)	0.0506 (4)	0.0556 (4)	0.0037 (2)	0.0072 (3)	0.0093 (2)
O1	0.0403 (9)	0.0630 (10)	0.0806 (11)	−0.0003 (7)	0.0080 (8)	0.0119 (9)
O2	0.0538 (10)	0.0559 (10)	0.0910 (13)	−0.0012 (8)	0.0182 (9)	0.0175 (9)
N1	0.0406 (10)	0.0458 (9)	0.0491 (9)	0.0004 (7)	0.0109 (7)	−0.0011 (8)
N2	0.0505 (11)	0.0558 (11)	0.0465 (10)	−0.0031 (8)	0.0101 (8)	−0.0021 (8)
N3	0.109 (2)	0.0805 (16)	0.0738 (14)	0.0062 (15)	0.0411 (14)	−0.0142 (13)
C1	0.0479 (16)	0.121 (3)	0.110 (2)	0.0012 (17)	0.0040 (16)	0.013 (2)
C2	0.0450 (14)	0.0844 (18)	0.103 (2)	−0.0084 (13)	0.0157 (14)	0.0109 (16)
C3	0.0440 (12)	0.0441 (12)	0.0618 (13)	0.0041 (9)	0.0147 (10)	−0.0015 (10)
C4	0.0422 (12)	0.0447 (11)	0.0694 (14)	0.0037 (9)	0.0121 (10)	0.0062 (10)
C5	0.0601 (14)	0.0567 (13)	0.0515 (12)	−0.0083 (11)	0.0186 (10)	−0.0062 (10)
C6	0.0595 (14)	0.0600 (13)	0.0433 (11)	−0.0119 (11)	0.0058 (10)	0.0036 (10)
C7	0.0379 (11)	0.0413 (11)	0.0460 (11)	−0.0057 (8)	0.0128 (8)	0.0061 (8)
C8	0.0664 (16)	0.0598 (14)	0.0473 (12)	−0.0082 (12)	0.0183 (11)	−0.0048 (11)

### Geometric parameters (Å, °)

**Table:**

S1—C7	1.736 (2)	C1—H1B	0.9600
S1—C6	1.811 (2)	C1—H1C	0.9600
O1—C3	1.325 (3)	C2—H2B	0.9700
O1—C2	1.460 (3)	C2—H2C	0.9700
O2—C3	1.198 (3)	C3—C4	1.507 (3)
N1—C7	1.334 (3)	C4—H4A	0.9700
N1—C4	1.446 (3)	C4—H4B	0.9700
N1—C5	1.460 (3)	C5—C6	1.508 (3)
N2—C7	1.309 (3)	C5—H5A	0.9700
N2—C8	1.322 (3)	C5—H5B	0.9700
N3—C8	1.145 (3)	C6—H6A	0.9700
C1—C2	1.459 (4)	C6—H6B	0.9700
C1—H1A	0.9600		
			
C7—S1—C6	91.34 (10)	N1—C4—H4A	109.3
C3—O1—C2	115.73 (19)	C3—C4—H4A	109.3
C7—N1—C4	120.76 (17)	N1—C4—H4B	109.3
C7—N1—C5	114.95 (17)	C3—C4—H4B	109.3
C4—N1—C5	121.17 (18)	H4A—C4—H4B	108.0
C7—N2—C8	118.10 (19)	N1—C5—C6	106.08 (18)
C2—C1—H1A	109.5	N1—C5—H5A	110.5
C2—C1—H1B	109.5	C6—C5—H5A	110.5
H1A—C1—H1B	109.5	N1—C5—H5B	110.5
C2—C1—H1C	109.5	C6—C5—H5B	110.5
H1A—C1—H1C	109.5	H5A—C5—H5B	108.7
H1B—C1—H1C	109.5	C5—C6—S1	105.78 (15)
C1—C2—O1	108.6 (2)	C5—C6—H6A	110.6
C1—C2—H2B	110.0	S1—C6—H6A	110.6
O1—C2—H2B	110.0	C5—C6—H6B	110.6
C1—C2—H2C	110.0	S1—C6—H6B	110.6
O1—C2—H2C	110.0	H6A—C6—H6B	108.7
H2B—C2—H2C	108.4	N2—C7—N1	121.22 (19)
O2—C3—O1	124.6 (2)	N2—C7—S1	126.07 (17)
O2—C3—C4	125.1 (2)	N1—C7—S1	112.71 (14)
O1—C3—C4	110.33 (18)	N3—C8—N2	174.8 (3)
N1—C4—C3	111.65 (18)		
			
C3—O1—C2—C1	178.6 (2)	C7—S1—C6—C5	21.72 (16)
C2—O1—C3—O2	1.4 (3)	C8—N2—C7—N1	177.09 (19)
C2—O1—C3—C4	−178.3 (2)	C8—N2—C7—S1	−3.9 (3)
C7—N1—C4—C3	81.0 (2)	C4—N1—C7—N2	6.6 (3)
C5—N1—C4—C3	−78.0 (2)	C5—N1—C7—N2	166.86 (19)
O2—C3—C4—N1	0.4 (3)	C4—N1—C7—S1	−172.54 (15)
O1—C3—C4—N1	−179.87 (18)	C5—N1—C7—S1	−12.3 (2)
C7—N1—C5—C6	28.7 (2)	C6—S1—C7—N2	174.48 (19)
C4—N1—C5—C6	−171.18 (17)	C6—S1—C7—N1	−6.41 (16)
N1—C5—C6—S1	−30.7 (2)		

### Hydrogen-bond geometry (Å, °)

**Table:**

*D*—H···*A*	*D*—H	H···*A*	*D*···*A*	*D*—H···*A*
C2—H2C···O2^i^	0.97	2.56	3.284 (3)	132
C4—H4B···O2^ii^	0.97	2.50	3.431 (3)	162

Symmetry codes: (i) −*x*+1/2, −*y*+1/2, −*z*; (ii) *x*, *y*+1, *z*.
